# Social cognition and social functioning in patients with amnestic mild cognitive impairment or Alzheimer’s dementia

**DOI:** 10.1111/jnp.12223

**Published:** 2020-09-26

**Authors:** Roy P. C. Kessels, Maaike Waanders‐Oude Elferink, Ilse van Tilborg

**Affiliations:** ^1^ Donders Institute for Brain, Cognition and Behaviour Radboud University Nijmegen The Netherlands; ^2^ Department of Medical Psychology and Radboudumc Alzheimer Center Radboud University Medical Center Nijmegen The Netherlands; ^3^ Vincent van Gogh Institute for Psychiatry Venray The Netherlands; ^4^ Department of Medical Psychology ZGT Hospital Almelo/Hengelo The Netherlands; ^5^ Mediant Mental Health Care Enschede The Netherlands

**Keywords:** social cognition, dementia, assessment, affective neuroscience, emotion, theory of mind

## Abstract

The aim of the present study was to examine social cognition and social functioning in a group of amnestic mild cognitive impairment (aMCI) and Alzheimer’s dementia (AD) patients. Thirty one people with aMCI, 29 individuals with AD, and 45 healthy older adults participated in the study. Facial expressions of happiness, anger, fear, disgust, and surprise presented in different intensities had to be labelled. Mentalizing was assessed using first‐order belief theory of mind (ToM) stories and everyday social functioning by the Inventory of Interpersonal Situations (IIS), completed by an informant. aMCI patients were impaired in recognizing the emotions anger, disgust, and fear, while AD patients were impaired in recognizing the emotions anger, disgust, and surprise. More importantly, no significant differences between aMCI and AD patients were found on overall emotion recognition. Both the aMCI and AD patients were impaired on the ToM task, but no differences between the aMCI and AD patients were found. On everyday social functioning, only the AD patients showed impairments. No associations between the IIS and ToM were found, but the IIS and emotion perception were significantly correlated. Regression analysis taking all potentially confounding variables into account showed that only mood, but not the social‐cognitive task performance or any other cognitive variable, predicted social functioning. aMCI and AD patients demonstrated impairments in mentalizing and facial emotion perception, and showed decrements in everyday social functioning. Informing caregivers about these deficits may help them to understand deficits in social cognition that may be present already in the MCI stage of Alzheimer’s disease.

## Background

Social cognition refers to a variety of cognitive processes that underly social interactions. Social cognition is argued to consist of several core components. These include the perception of information that is socially relevant (for instance emotional facial expressions displayed by others), and the ability to ‘mentalize’ (also referred to as Theory of Mind [ToM]), that is to infer thoughts, intentions, and feelings of oneself and others. Combined with knowledge on social rules and roles, these processes are required for everyday social functioning (Roelofs, Wingbermühle, Egger & Kessels, 2017). Consequently, there is evidence that disruptions in social‐cognitive processes, for instance as a result of neurodegenerative disease, may hamper an individual’s social interaction and thus negatively affect interpersonal function (Christidi, Miglaccio, Santamaría‐García, Santangelo & Trojsi, 2018). In some neurodegenerative syndromes, such as frontotemporal dementia, social‐cognitive deficits are considered a hallmark of the clinical syndrome, especially in its behavioural variant, which play an important role in the development of the behavioural changes seen in these patients (e.g. Gregory *et al*., 2002; Shany‐Ur & Rankin, 2011).

Social cognition in patients with Alzheimer’s dementia (AD) has been studied less extensively than in FTD, possibly because episodic memory and orientation difficulties are the most prominent deficits in AD, potentially overshadowing social‐cognitive impairment. However, AD patients may also show impairments in social behaviour, emotion perception, and ToM (Christidi *et al*., 2018; Savva *et al*., 2009). Furthermore, these symptoms may be more disturbing than the cognitive changes and have been associated with a reduced quality of life, increased caregiver burden, and increased cost associated with dementia care (Moore, Zhu, & Clipp, 2001; Spitzer, Shafir, Lerman, & Werner, 2019). Moreover, deficits in social cognition, notably emotion perception and ToM, may already be present in the pre‐dementia stage (Bora & Yener, 2017), referred to as (amnestic) mild cognitive impairment (aMCI; Albert *et al*., 2011).

With respect to the assessment of emotion perception, most studies in AD have studied labelling of static, full‐blown facial expressions using the six basic emotions – happiness, sadness, surprise, anger, fear, and disgust (Ekman, 1992). A systematic review by Torres Mendonça De Melo Fádel, Santos De Carvalho, Belfort Almeida Dos Santos, and Dourado (2019) showed that impairments in the perception and labelling of facial expressions are not consistently found in AD. They argue that the lack of standardized assessment instruments and the heterogeneity of the methods and samples used across studies hamper comparisons. In addition, a review by Waanders‐Oude Elferink, van Tilborg, and Kessels (2015) on emotion perception in AD and MCI (both its amnestic and non‐amnestic subtype) showed that very few studies have examined the effect of varying the intensities of facial emotional expressions. This is relevant, as using full‐blown emotional expressions may lack sensitivity and result in a near‐ceiling performance on some emotions (notably happy facial expressions; Kessels *et al*., 2014; Torres Mendonça De Melo Fádel *et al*., 2019) that may obscure actual deficits compared to older controls, notably in amnestic MCI patients. Indeed, Spoletini *et al*. (2008) showed in their research that patients with aMCI only exhibit difficulties at lower emotional intensities. These findings have been replicated in a study showing that individuals with aMCI performed at the level of individuals with mild AD when emotional expression was more subtle (until 40%), whereas aMCI patients performed at the level of cognitively unimpaired controls at higher intensities of emotional expression (80–100%; Bediou *et al*., 2009). One complicating factor is that the interpretation of study results may be hampered by other, non‐social‐cognitive deficits that are by definition present in aMCI and AD patients (Shany‐Ur & Rankin, 2011). Furthermore, preserved perception of specific dynamically presented emotions (e.g. disgust) has also been reported in AD (Henry *et al*., 2008).

Considering ToM, previous studies in patients with AD have shown less consistent results than those on emotion perception, findings which are also complicated to interpret because of impairments in memory and executive functions (e.g. Elamin, Pender, Hardiman, & Abrahams, 2012; McCade, Savage, & Naismith, 2011). Within ToM paradigms, a distinction is often made between first‐order beliefs (inferring what others think) and second‐order beliefs (inferring what others think about others). Several studies investigating ToM abilities in AD patients have primarily reported deficits on second‐order belief ToM tasks, which are usually presented as a story narrative (Castelli *et al*., 2001; Cuerva *et al*., 2001; Gregory *et al*., 2002; Sandoz, Démonet, & Fossard, 2014). However, second‐order mentalizing tasks clearly place heavy demands on working memory and executive function, rendering the interpretation of the results of these studies difficult (Elamin *et al*., 2012). Indeed, there is evidence that the basic ability involved in ToM (i.e. making inferences about beliefs held by another person) remains intact under less complex task conditions in patients with AD, as is the case in first‐order beliefs (El Haj, Gély‐Nargeot, & Raffard, 2015; Kemp, Després, Sellal, & Dufour, 2012). Studies of ToM in the early stages of AD or in individuals with aMCI are limited. The meta‐analysis by Bora and Yener (2017) only identified two studies that distinguished first‐ and second‐order false belief tasks, with impairments only being found in second‐order false belief tasks. Compared to FTD, ToM deficits in AD and aMCI are typically less pronounced (cf. Bora, Velakoulis, & Walterfang, 2016; Gossink *et al*., 2018).

To date, studies directly comparing aMCI patients, AD patients, and older controls without cognitive impairment on both aspects of social cognition, that is emotion perception and ToM, are scarce. Furthermore, the extent to which social‐cognitive deficits affect everyday life interpersonal functioning has received little attention in general. A recent meta‐analysis on autism spectrum disorders (ASD; Trevisan & Birmingham, 2016) showed correlations between emotion perception and observed measures of receptive and expressive communication and interpersonal skills during social interaction in ASD, also stressing that only limited research has been done in that field. In aMCI, we only found one study (McCade, Savage & Naismith, 2011) that examined emotion perception and daily social functioning (measured with The World Health Organization Disability Assessment Schedule II (WHODAS), failing to demonstrate an association between the two. However, they found that increased caregiver burden was associated with worse recognition of angry expressions in aMCI patients. A study in AD patients (Shimokawa *et al*., 2001) showed that deteriorating emotion recognition, rather than overall cognitive decline, negatively affected the interpersonal behaviour of AD patients. Poveda, Osborne‐Crowley, Laidlaw, Macleod, and Power (2017) also concluded that individuals with AD were impaired at emotion recognition measured using video vignettes (already in the early disease stages), which could not be accounted for by general cognitive decline. However, no relation was found between measures of social cognition and the Birmingham Relationship Continuity Measure (BRCM) that assesses the caregiver’s perceived relationship with the person with dementia.

The first aim of the present study was to investigate social cognition in aMCI and AD patients, and compare their performance to an older control group. We examined the perception of facial emotional expressions presented as morphs, gradually expressing one of the six basic emotions from neutral to four levels of intensity, which may be a more sensitive measure than using static emotional expressions (Kessels, Montagne, Hendriks, Perrett, & De Haan, 2014). We hypothesized that aMCI patients are especially impaired at lower intensities, while AD patients show impairments on the higher‐intensity emotions as well, compared to healthy controls. Furthermore, we hypothesized to find more pronounced deficits in the perception of negative emotions (i.e. sadness, fear, anger, and disgust), as these are generally less easily recognized than non‐negative emotions (happiness and surprise), even in healthy individuals (Fernandez‐Duque & Black, 2005). By directly comparing the performance of AD patients and aMCI patients with older controls using different intensity facial expressions, more subtle emotion perception deficits may possibly be identified than in previous studies. The second aim was to examine ToM ability in aMCI and AD, hypothesizing that second‐order false beliefs are especially compromised in aMCI and AD, with AD performing overall worse than aMCI patients on ToM ability. While these two aims may replicate previous findings, our third aim clearly extends previous work, that is, to examine the extent to which ToM and facial emotion perception performance are associated with everyday social functioning. Also, we took into account the performance on working memory and executive function, as well as mood, which may affect performance on the social‐cognitive tasks (cf. Shany‐Ur & Rankin, 2011). We hypothesized that a worse performance on ToM and emotion perception is associated with impairments in everyday social functioning, both in aMCI and AD.

## Methods

### Participants

Thirty‐one aMCI patients with a mean age of 75.4 (*SD* = 6.6) and 29 AD patients with a mean age of 76.8 (*SD* = 6.5) were recruited from the department of medical psychology of Ziekenhuis Groep Twente in Almelo, the Netherlands, a general hospital, and Mediant in Enschede, the Netherlands, a mental health care facility. The clinical diagnoses were made in a multidisciplinary setting by a team of neurologists, geriatricians, and neuropsychologists using the results of extensive neuropsychological testing covering all cognitive domains, a clinical interview with the patient and caregiver for the assessment of daily functioning, medical and psychiatric history taking as well as other important (biographical) information, laboratory investigations, neurological examination, and neuroimaging data (MRI). AD patients met the National Institute on Aging and the Alzheimer’s Association criteria for dementia due to Alzheimer’s disease (McKhann *et al*., 2011), and all were in the mild to moderate stage (Clinical Dementia Rating [CDR] 1–2). The amnestic MCI patients (CDR 0.5) met the National Institute on Aging and the Alzheimer’s Association criteria for single‐domain amnestic mild cognitive impairment (Albert *et al*., 2011). Mean performance on the Mini‐Mental State Examination (MMSE; Folstein, Folstein, & McHugh, 1975) was 19.2 (*SD* = 4.7) for the AD patients and 24.2 (*SD* = 2.8) for the aMCI patients.

Forty‐five healthy older adults served as controls, with a mean age of 72.8 (*SD* = 6.0). Healthy controls (HC) were participants without cognitive impairments who visited the memory clinic or volunteers. None of the control participants had a history of neurological or psychiatric disease, and their mean performance on the MMSE was 28.0 (*SD* = 2.8). Education level was categorized for all participants as low (only primary education or less), average (basic vocational training), or high (advanced vocational or academic training) in accordance with the Dutch educational system that is comparable with the International Standard Classification of Education (UNESCO, 2011; Van der Elst, Van Boxtel, Van Breukelen & Jolles, 2005). Table 1 shows the characteristics for the participants. A slight, yet statistically significant age difference was found between the groups (*F*(2,102) = 3.6, *p* = .03), with the AD patients being on average 3.9 years older than the controls (*p* = .032). No group differences were found on sex distribution (χ^2^(2) = 2.4, *p* = .30). With respect to education level, the AD group had the lowest educational achievement compared to the other groups (Kruskal–Wallis *H*(2) = 10.9, *p* = .004).

**Table 1:** Characteristics (mean ± standard deviation) for the patients with amnestic mild cognitive impairment (aMCI), Alzheimer’s dementia (AD), and the controls

	AD (*N* = 29)		aMCI (*N* = 31)		Controls (*N* = 45)	
	*M*	*SD*	*M*	*SD*	*M*	*SD*
Age	76.8*	6.5	75.4	6.6	72.8	6.0
Sex (*N* m:f)	11:18		18:13		22:23	
Education level (*N*)						
Low	10		8		9	
Average	18		18		19	
High	1*		5		17	
MMSE	19.2*^#^	4.7	24.2*	2.8	28.0^#^	2.8
GDS‐30	6.8	4.6	6.0	3.0	6.3	6.1
Digit Span WAIS‐III	10.5*^#^	2.3	12.5*	2.5	14.4	3.6
CORVIST‐FP	7.1	0.8	7.0	0.9	7.2	0.9
CAMCOG‐R Executive score	9.1*^#^	4.3	14.7	5.1	15.1	6.8

Note

CAMCOG‐R = Revised Cambridge Cognitive Examination; CORVIST‐FP = Cortical Vision Screening Test – Face Perception subtest; GDS‐30 = Geriatric Depression Scale; MMSE = Mini‐Mental State Examination; NART = National Adult Reading Test.

* *p *< .05 compared to control group; ^#^ *p *< .05 compared to aMCI.

Also, informants were asked to rate the patients’ or the healthy controls’ social abilities; these were recruited through participant nomination and were most likely to be the patient’s or control’s spouse or child (>95% of informants fell into one of these two categories). This study was approved by the Institutional Review Board of Ziekenhuis Groep Twente, and written informed consents were obtained in all participants according to the Declaration of Helsinki.

## Materials

### Emotion perception

The Emotion Recognition Task (ERT; Kessels *et al*., 2014) was used to measure the perception of facial emotional expressions using the six universal emotions: anger, sadness, surprise, happiness, fear, and disgust. In this test, emotional facial expressions are presented as morphs gradually expressing one of the six basic emotions from neutral to four levels of intensity (40%, 60%, 80%, and 100%). In each trial, the displayed emotion has to be labelled using a six alternative forced choice response with no time restriction. The faces were presented beginning with the lowest intensities. Following three practice trials, the different types of emotion of the same intensity were presented in pseudo‐random, fixed order to control for possible order effects of previously encountered emotion types that may influence the response. The test contains 96 items, and its average duration is 15 min.

### Theory of Mind

Mentalizing was examined by four first‐order belief ToM stories that were developed in a previous study (Oosterman, de Goede, Wester, van Zandvoort, & Kessels, 2011). For each story, a factual question and a first‐order belief ToM question were available. For the ToM questions, participants had to infer the feelings, ideas, or knowledge of a character in the story. The factual questions asked details that could be literally abstracted from the text requiring no inferential reading, to control for possible confounding factors such as difficulties in reading or memory that may adversely affect task performance. The questions connected to each story were independent: the correct answer to one question was not part of the reasoning process with respect to the other question. For example: ‘Carl is going to buy a car. Carl thinks a Volkswagen is much nicer than a Honda. The Volkswagen is too expensive and he buys the Honda for 5000 Euros. When he walks past a car dealer the following week, he sees a Volkswagen for 4000 Euros. Carl regrets.’ The factual question is: ‘Which car did Carl buy?’ and the ToM question is: ‘What does Carl regret?’

All participants had to read each story out aloud from a single page. Next, two questions had to be answered (one first‐order ToM and one factual question). The page containing the story was always present and participants were allowed to look at it when answering questions to minimize memory burden. One point was awarded for a fully correct answer, 0.5 points for a partially correct answer, and 0 for an incorrect or no answer; the maximum score was four points.

### Social functioning

Informants were asked to rate the patients’ or the healthy controls’ social abilities by adapting the Inventory of Interpersonal Situations (IIS; Dam‐Baggen & Kraaimaat, 1999) for the purpose of this study. This inventory consists of 35 items formulated as responses to specific everyday social situations (e.g. ‘Joining a conversation of a small group of people’, ‘Asking a friend to go out with you’). The original IIS provides scores for both a Discomfort and a Frequency scale. In our study, we only used the Frequency scale as an index of social functioning. We adjusted the original self‐report instruction of the IIS to a third‐party rating instruction. The informant had to rate how often certain behaviour occurred using a 5‐point rating scale. The IIS includes five subscales: Giving criticism, Expressing opinion, Giving compliments, Initiating contacts, and Positive self‐avaluation.

### Cognitive variables and mood

For descriptive purposes and to perform correlational analyses, several other measures were administered. In addition to the MMSE, participants completed the executive function subtests of the Revised Cambridge Cognitive Examination (CAMCOG‐R; Roth *et al*., 1986). The Digit Span subtest from the Wechsler Adult Intelligence Scale – Third Edition (WAIS‐III; Wechsler, 1997) was administered to measure working memory. The Face Perception subtest of the Cortical Vision Screening Test (CORVIST; Plant & Warrington, 2001) was used to detect impairment in the facial perception. In addition, patients completed the Geriatric Depression Scale to measure depressive (GDS‐30; Yesavage *et al*., 1982–1983). Table 1 summarizes the performance of the participants on these tasks.

## Analyses

The performance on the ERT was analysed using a multivariate general linear model (GLM) repeated‐measure analysis with Emotion Type (six levels: anger, happiness, surprise, fear, sadness, and disgust) and Emotion Intensity (four levels: 40%, 60%, 80%, and 100%) as within‐subject factor and Group (AD, aMCI, and HC) as between‐subject factor. Subsequently, repeated‐measure GLM analyses were performed for the six emotions separately (with Emotion Intensity as within‐subject factor and Group as a between‐subject factor) with Fisher’s least significant difference (LSD) post‐hoc analyses.

As the ToM outcome measures were not normally distributed, these measures were compared between the groups using Kruskal–Wallis test, with ToM score as dependent variable and Group (aMCI, AD, and HC) as independent variable. Subsequently, pair‐wise comparisons with Kruskal–Wallis tests were used as follow‐up analysis to further examine differences between the individual groups. Social functioning was analysed using one‐way ANOVA for each IIS subscale and the IIS total score with Group as between‐subject factor, with Fisher’s LSD post‐hoc analyses.

Subsequently, Spearman correlation coefficients were calculated between ToM performance, facial emotion perception, and the IIS total score and its subscales. Because ToM tests are mentally demanding and performance may be impaired due to executive deficits or overall cognitive impairment, we also calculated the Spearman correlation coefficients between ToM performance and Digit Span and the CAMCOG‐R executive score. Also, the correlation between mood and social cognition was examined using Spearman’s ρ. Next, we performed a linear regression analysis with the cognitive tasks, mood, ERT, and ToM as predictors of social functioning.

For the analyses of variance, effect sizes were calculated (η*_p_* ^2^ or Cohen’s *d*) and α was set at .05. The correlations were computed for all groups taken together and here α was set at .01 to reduce the risk of type I errors.

## Results

### Emotion perception

**Figure 1:**
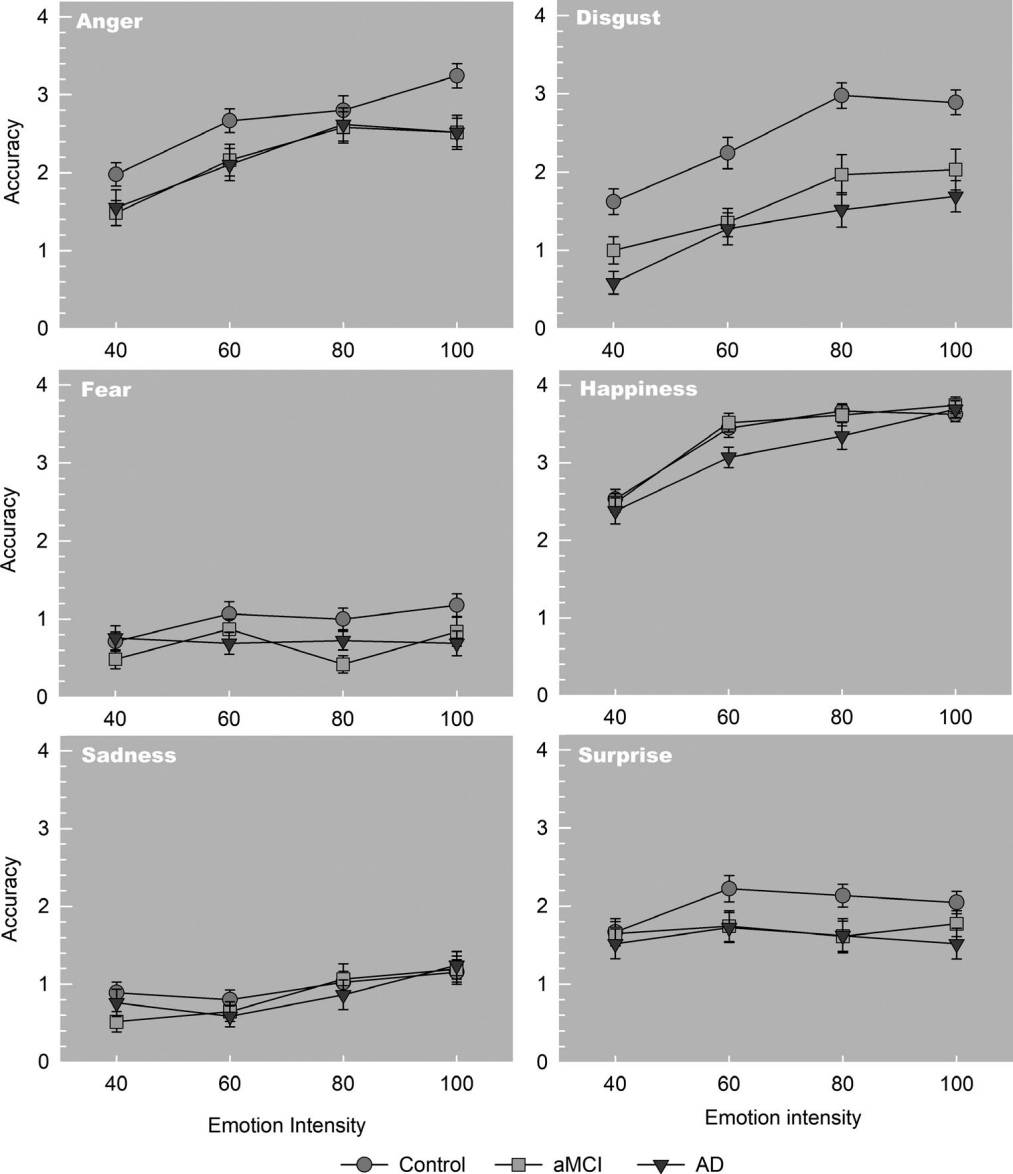
Mean performance (number of correctly identified emotions, max = 4; ± *SEM*) of the patients with amnestic mild cognitive impairment (aMCI), Alzheimer’s dementia (AD), and the healthy controls on the different emotional intensities (40–60–80–100%) for the six different emotions.

Figure 1 shows the results on the ERT for the different emotions and intensities for the three groups. There was a main effect of Group (*F*(2,102) = 15.0, *p* < .001, η*_p_* ^2^ = 0.23), with both the aMCI (*p* < .001) and the AD patients (*p* < .001) performing overall worse than the HC. The repeated‐measure analyses revealed a main effect of Emotion Type (*F*(5,98) = 262.6, *p* < .001, η_p_^2^ = 0.93), and Emotion Intensity (*F*(3,100) = 77.83, *p* < .001, η*_p_* ^2^ = 0.70). This indicates overall performance differences across the six emotions and that overall performance improved when faces with a higher level of emotion intensity were presented. A significant interaction effect was found for Emotion Type × Group (*F*(10,198) = 2.61, *p* = .005, η_p_^2^ = 0.12), indicating that the performance on the different emotions differed across groups. The interaction for Emotion Type × Emotion Intensity × Group (*F*(30,178) = 1.2, *p* = .20, η*_p_* ^2^ = 0.17), and for Emotion Intensity × Group (*F*(6,202) = 0.6, *p* = .75 η*_p_* ^2^ = 0.02), was not statistically significant, indicating that different intensities of facial expressions did not have a differential group effect on performance. Post‐hoc tests showed that the aMCI and AD groups performed worse than the controls (*p* < .001), but there were no differences in performance between the aMCI and AD groups (*p* = .33, η*_p_* ^2^ = 0.021).

Separate analyses for each emotion revealed a Group effect for the emotions anger (*F*(2,102) = 4.2, *p* = .017, η*_p_* ^2^ = 0.08), disgust (*F*(2,102) = 18.7, *p* < .001, η_p_^2^ = 0.27), fear (*F*(2,102) = 3.5, *p* = .034, η*_p_* ^2^ = 0.06), and surprise (*F*(2,102) = 4.20, *p* = .018, η*_p_* ^2^ = 0.08). Post‐hoc tests showed that AD patients performed worse on recognizing anger (*p* = .019, *d* = 0.62), disgust (*p* < .001, *d* = 1.43), and surprise (*p* = .009, *d* = 0.56) in comparison to HC. aMCI patients performed worse on anger (*p* = .014, *d* = 0.62), disgust (*p* < .001, *d = *0.95), and fear (*p* = .017, *d* = 0.52), compared to HC.

### Theory of Mind

**Figure 2:**
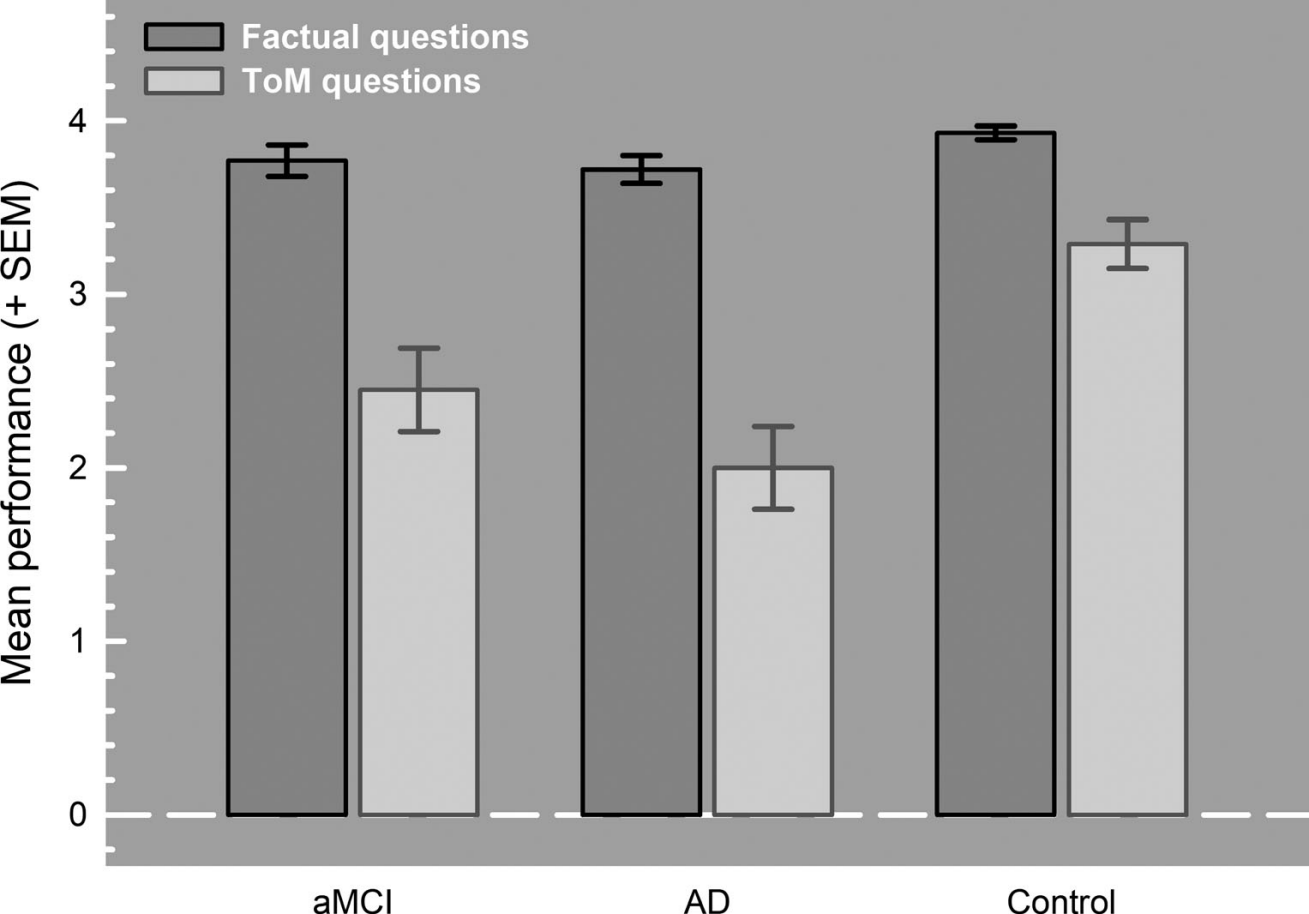
Performance (mean ± *SEM*) on the theory of mind task of the patients with amnestic mild cognitive impairment (aMCI), Alzheimer’s dementia (AD), and the healthy controls.

Figure 2 shows the results on the ToM task and Table 2 shows the results on the IIS. No significant differences were found between AD patients, aMCI patients and HC on the factual questions (*H*(2) = 5.91, *p* = .052, η*_p_* ^2^ = .057). Significant differences were found between the groups on the ToM questions (*H*(2) = 18.74, *p* < .005, *d* = 0.89). Pair‐wise comparisons (Mann–Whitney *U* test) showed that the aMCI patients’ accuracy on the ToM questions was lower than the HC group (*U* = 446.5, *p* = .005, *d* = 0.64). Also, the AD patients were less accurate than healthy controls on the ToM questions (*U* = 290.0, *p* < .001, *d* = 1.06). ToM accuracy in the aMCI patients did not differ from that of the AD patients (*U* = 367.0, *p* = .21, *d* = 0.32).

**Table 2:** Mean scores (and standard deviations) of the Inventory of Interpersonal Situations (IIS) and its subscales for the patients with amnestic mild cognitive impairment (aMCI), Alzheimer’s dementia (AD), and the healthy control group

	AD		aMCI		Controls	
	*M*	*SD*	*M*	*SD*	*M*	*SD*
IIS Total score	93.97*	22.18	103.16	23.19	107.13	20.24
Giving criticizing	15.21	4.48	15.48	6.05	16.71	4.87
Expressing opinions	14.59*	4.21	16.68	4.27	17.38	4.27
Giving compliments	13.31	4.29	15.00	3.62	15.33	3.23
Initiating contacts	14.10*	3.91	15.32	4.59	16.33	2.98
Positive self‐evaluation	12.48	3.98	13.81	3.29	14.00	2.59

Note

* *p* < .05 compared to healthy controls.

### Social functioning

With respect to the ratings on the IIS, a significant main effect of Group was found on the IIS total score (*F*(2,101) = 3.3, *p* = .040, η*_p_* ^2^ = .062), the IIS subscales Expressing opinions (*F*(2,101) = 3.8, *p* = .025, η*_p_* ^2^ = .071), and Initiating contacts (*F*(2,101) = 3.2, *p* = .046, η*_p_* ^2^ = .059). Post‐hoc tests showed significantly lower ratings for AD patients compared to the controls on the IIS total score (*p* = .012, *d* = 0.62), and the subscales Expressing opinions (*p* = .07, *d* = 0.66) and Initiating contacts (*p* = .015, *d* = 0.64). The aMCI patients neither significantly differed from the AD patients nor from the healthy controls. No group main effects were found for Giving criticism, Giving compliments, and Positive self‐evaluation (all *F*‐values < 2.9).

### Correlational and regression analyses

First, we examined the relation between emotion perception and ToM, and social functioning (data not shown). Here, we did not find any significant associations between the performance on the ToM task and the IIS total score or any of its subscales (all ρ < 0.249). Only the ERT total score was borderline significantly correlated with the IIS total score (ρ = .25, *p* = .01).

Table 3 shows the Spearman correlation coefficients between the social‐cognitive tasks, the neurocognitive tasks, and mood. We examined the relation between the social‐cognitive measures and the other cognitive tasks and mood. Here, general cognitive function (MMSE) correlated with ToM (ρ = .46, *p* < .005), the IIS Total Score (ρ = 0.27, *p* = .004), Initiating contacts scale (ρ = 0.30, p = .002), and ERT Total score (ρ = 0.37, *p* < .001) and Disgust (ρ = 0.47, *p* < .005). Working memory correlated with ToM (ρ = 0.31, *p* = .002), IIS Total Score (ρ = 0.31, *p* < .001), Giving compliments (ρ = 0.26, *p* = .008) and Initiating contacts (ρ = 0.39, *p* < .0005), and ERT Total score (ρ = 0.30, *p* = .002), Disgust (ρ = 0.28, *p* = .004), and Happiness (ρ = 0.25, *p* = .01). Executive function correlated significantly with ERT Total score (ρ = 0.29, *p* = .003) and Surprise (ρ = 0.27, *p* = 0.006). Mood was negatively correlated with the IIS Total score (ρ = −.33, *p* < .001), Initiating contacts (ρ = −.36, *p* < .001) and Positive self‐evaluation (ρ = 0.27, *p* = .006), and with the ERT emotion Happiness (ρ = −0.27, *p* = .005). Finally, ToM correlated significantly with ERT Total score (ρ = 0.25, *p* = .01), Disgust (ρ = 0.30, *p* = .002), and Happiness (ρ = 0.35, *p* < .001).

**Table 3:** Spearman ρ correlation coefficients between the social‐cognitive tasks and the neurocognitive tasks and mood

	MMSE	Working memory	Executive functioning	GDS‐30	ToM
ToM	.46**	.31**	.05	−.19	
IIS Total score	.27**	.31**	−.03	−.33**	.09
Giving criticism	.18	.18	−.06	−.21	.19
Expressing opinions	.21	.17	.10	−.18	.01
Giving compliments	.20	.26**	−.06	−.18	.05
Initiating contacts	.30**	.39**	−.01	−.36**	.04
Positive self‐evaluation	.16	.22	−.10	−.27**	.02
ERT total score	.37**	.30**	.29**	.00	.25**
Anger	.16	.25	.20	.04	.12
Disgust	.47**	.28**	.27	−.03	.30**
Fear	.09	.12	−.04	.11	.01
Happiness	.25	.25**	.03	−.27**	.35**
Sadness	.03	.11	.11	.06	.15
Surprise	.13	−.05	.27**	.15	.01

Note

GDS = Geriatric Depression Scale (30‐item version); MMSE = Mini‐Mental State Examination; IIS = Inventory of Interpersonal Situations; ERT = Emotion Recognition Task; ToM = Theory of Mind questions.

** *p* < .01.

Finally, a linear regression analysis with IIS total score as dependent variables and the cognitive measures, mood, emotion perception, and ToM as predictors resulted in an overall statistically significant model (*R* ^2^ = 0.17, *F*(6,95) = 3.33, *p* = .005; checked for multicollinearity), but revealed that only mood was a significant predictor of social functioning (β = −0.30, *p* = .002; see Table 4).

**Table 4:** Regression results (standardized β, *t,* and *p*‐values) for the cognitive variables, mood, and emotion perception and theory of mind (ToM) as predictors of social functioning as measured with the Inventory of Interpersonal Situations’ total score

Predictor	β	*t*‐value	*p*‐value
MMSE	0.120	1.02	0.31
Executive function	−0.007	−0.07	0.94
Working memory	0.135	1.20	0.24
GDS‐30	−0.304	−3.11	0.002
ToM questions	−1.113	−1.05	0.30
ERT total score	0.128	1.21	0.23

Note

MMSE = Mini‐Mental State Examination; GDS‐30 = Geriatric Depression Scale (30‐item version), ERT = Emotion Recognition Task.

## Discussion

In this study, we set out to examine social cognition (i.e. emotion recognition and mentalizing) and everyday social functioning as rated by significant others in aMCI and AD patients, compared to cognitively unimpaired older adults. First, we demonstrated that AD patients had impairments in the recognition of the emotions anger, disgust, and surprise, while aMCI patients performed worse than controls on anger, disgust, and fear. The intensities of the presented facial expressions did not interact with the performance across the patient groups, indicating that the performance was not only impaired for the lower intensities, but also for the higher (i.e. full‐blown) intensities. Non‐emotional face perception ability did not differ across the three groups. Second, we showed that aMCI and AD patients performed worse on answering questions requiring the ability to infer the thoughts and feelings of others (mentalizing or ToM) in theory of mind ability, but did not find any differences in ToM performance between the two patient groups. The performance on answering factual questions (i.e. not requiring mentalizing) did not significantly differ between the groups. Thus, the results are not due to difficulty in reading or comprehension of the stories. Third, we found that AD, but not aMCI, patients were impaired in everyday social functioning compared to controls, as measured using a third‐party rating scale (the IIS), a brief inventory developed to measure current social skills. aMCI patients did not significantly differ in observed social functioning compared to healthy controls (or to AD patients). Finally, we examined the correlation between emotion perception, mentalizing ability, and observed social functioning. Here, a significant association was found between facial emotion perception and social functioning. No significant association could be determined between ToM and social functioning. Also, the two aspects of social cognition, ToM and emotion recognition, were significantly correlated. However, taking all potentially confounding variables into account revealed that only mood was a significant predictor of social functioning.

Our results extend those of previous studies, in which mostly deficits in emotion perception of negative emotions were found (see Waanders‐Oude Elferink, Van Tilborg, & Kessels, 2015, for a review). In our study, AD patients performed worse than HC on the emotions anger, disgust, and surprise. This is in line with other studies which also found worse performances in AD patients for the emotions anger (Bediou *et al*., 2009; Bertoux *et al*., 2015; Fujie *et al*., 2008; Maki, Yoshida, Yamaguchi & Yamaguchi, 2013; Philips, Scott, Henry, Mowat, & Bell, 2010; Spoletini *et al*., 2008), disgust (Bertoux *et al*., 2015; Drapeau, Gosselin, Gagnon, Perets & Lorain, 2009; Hargrave, Maddock, & Stone, 2002; Phillips *et al*., 2010; Spoletini *et al*., 2008; Wiechtek Ostos, Schenk, Baenziger, & von Gunten, 2011), and surprise (Bediou *et al*., 2009; Hargrave *et al*., 2002; Maki *et al*., 2013; Phillips *et al*., 2010). aMCI patients performed worse on the emotions anger, disgust, and fear compared to healthy controls. While several studies also found a selective deficit for the emotions anger (Fujie *et al*., 2008; McCade *et al*., 2013), sadness (Fujie *et al*., 2008), or fear (Richard‐Mornas *et al*., 2012; Spoletini *et al*., 2008) in aMCI, others did not find any differences between aMCI patients and controls on emotion perception (Bediou *et al*., 2009; Teng *et al*., 2007; Varjassyová *et al*., 2013; Weiss *et al*., 2008). We did not observe significant differences between AD patients, aMCI patients, and healthy older adults on recognizing happiness and sadness. This is in line with most previous studies that also did not demonstrate any deficits in recognizing happy faces in AD (Elamin *et al*., 2012; McLellan *et al*., 2008) and/or aMCI (Bora & Yener, 2017; McCade, Savage, & Naismith, 2011). The finding that AD and amnestic MCI patients did not significantly differ from healthy older adults on recognizing happy faces may be the result of differences in sensitivity across the different emotions rather than its positive valence, as happiness is commonly found to be the easiest of the six emotions to be labelled correctly (Kessels *et al*., 2014).

We did not find performance differences on the ERT between the aMCI and AD groups (the small effect size indicating that this in unlikely the result of limited power). So far, only a few studies have directly compared amnestic MCI patients with AD patients on measures of emotion perception with different intensities (Bediou *et al*., 2009; Spoletini *et al*., 2008; Weiss *et al*., 2008). Bediou *et al*. (2009) studied morphed emotional expressions using an (unreferenced) extended version of the task we used in the current study (Montagne *et al*., 2007) in a small sample of aMCI (*N* = 10) and AD (*N* = 10) patients, not finding significant differences between both groups, and only showing a worse performance of the AD patients on anger and the total ERT score compare to controls. However, the authors acknowledge that their sample size was too small to draw any firm conclusions. Spoletini *et al*. (2008) studied 50 aMCI patients and compared them to a sample of 50 AD patients and healthy controls on the Penn Emotion Recognition Test which includes high‐ and low‐intensity emotional expressions, showing that the AD patients differed from the aMCI and control groups on all five included emotions (surprised expressions were not included), but that the aMCI patients did not differ from controls. Weiss *et al*. (2008) examined 21 single and 30 multiple‐domain aMCI patients and compared them to 30 mild and 23 moderate AD patients using the Penn Emotion Recognition Test, but included only four emotions: happiness, anger, sadness, and fear. Separating single‐ and multiple‐domain aMCI patients they showed unimpaired emotion perception in the single‐domain patients, but impairments on sadness and fear (as well as overall emotion perception) in the multiple‐domain patients. Sadness and fear were impaired in both the mild and the moderate AD patients, and the moderate AD patients also were impaired at recognizing happy faces.

With respect to mentalizing ability, some authors have hypothesized that deficits in ToM tasks in cognitively impaired patients are due to the task’s cognitive demands, rather than to a mentalizing impairment as such (Choong & Doody, 2013). We indeed found a correlation between ToM performance and global cognitive functioning as measured by the MMSE, as well as working memory. However, in contrast to previous studies (see Sandoz *et al*., 2014), we did not find a significant correlation between executive functioning and ToM ability and also made sure the ToM task was not too difficult by only including first‐order belief questions. Several other studies also demonstrated a lack of association between ToM abilities and executive functions, for example in patients with the behavioural variant of frontotemporal dementia (Gregory *et al*., 2002; Lough, Gregory, & Hodges, 2001). Cavallini, Lecce, Bottiroli, Palladino, and Pagnin (2013) argued that these discrepancies may be due to the type of task used. This is also illustrated in a review by Sandoz *et al*. (2014) showing that depending on the task used (ToM stories vs. false belief paradigms), different executive abilities may be involved. We were only able to administer a short and global executive function measure (the executive function subtests of the CAMCOG‐R), which does not allow for isolating specific executive subprocesses. The extent to which executive functions and theory of mind abilities constitute overlapping or separate functions remains to be studied in more detail.

In contrast to previous studies (Bora & Berk, 2015), we did not find an association between ToM and depressive symptoms. One explanation for this finding may be that our sample reported relatively low levels of depressive symptoms. Our study did not find a correlation between ToM performance and everyday social interaction as observed by informants (i.e. the patient’s informal caregiver or spouse), but we did find a relation between emotion perception and everyday functioning. However, our regression model showed that only mood was a significant predictor of social functioning taking all social‐cognitive tasks into account in one model. This lack of a significant association between social‐cognitive tasks and social functioning may have partly been due to methodological factors. Social functioning was assessed using a third‐party rating. One could argue that self‐assessment may provide a more accurate evaluation of a patient’s emotional response, although this creates its own challenges because of the severe cognitive deficits and the lack of insight, especially in AD patients (Mullen, Howard, David, & Levy, 1996). In turn, ratings of social functioning by caregivers may also be biased by the nature of the current relationship with the patient, stress levels, and care burden experienced by caregivers (Clare *et al*., 2012).

Strengths of this study include the careful selection of participants, taken overall cognition, mood, and face perception into account. Also, we used an emotion recognition paradigm in which gradually increasing emotional intensities were presented. Although presenting less intense emotional expressions may have increased the ERT’s overall sensitivity to impairments compared to tasks that only used full‐blown expressions, we did not find evidence for deficits only on lower intensities in our samples (as evidenced by the nonsignificant interactions between group and intensity). A limitation is the study’s cross‐sectional design. Longitudinal follow‐up of performance during the course of aMCI and AD may improve the understanding of emotion processing deficits in these patient groups. Furthermore, although we feel that the scoring method for the ToM task is unambiguous, it was scored by only one rater, making it not possible to examine its inter‐rater reliability. Also, the AD group included more individuals with a low education than the other groups, despite our efforts to match the groups. However, adding education level as a covariate (data not shown) did not change any of our results. Finally, the use of the IIS can be criticized, as this scale was initially developed for measuring social participation in the context of social anxiety, although the use of its frequency rating scale provides a valid index of social participation in daily life. Recently, Sommerlad, Singeleton, Jones, Banerjee, and Livingstone (2017) developed the Social Functioning in Dementia Scale (SF‐DEM) that can be used to assess social functioning in dementia specifically. We recommend using this instrument, which was not yet available at the start of this study, in future research on social cognition in MCI and AD.

In all, our findings show that patients with aMCI and AD show impairments in emotion perception. Also, mentalizing or ToM deficits (first‐order beliefs) are already present in the aMCI stage of Alzheimer’s disease, stressing that the assessment of social‐cognitive abilities should be part of early cognitive assessment (see also Bora & Yener, 2017). Psychoeducation about altered behaviour and social deficits in the early stages of Alzheimer’s disease may also be important to optimize the patients’ and caregivers’ quality of life, as it may help them to understand and possible even reduce social interaction problems, diminishing caregiver burden. Further study is required to examine the relationship between social cognition and behavioural problems in everyday life.

## Funding

The authors received no financial support for the research, authorship, and/or publication of this article.

## Conflicts of interest

The authors declared no potential conflicts of interest with respect to the research, authorship, and/or publication of this article.

## Author contributions

**Roy P. C. Kessels** (Conceptualization; Formal analysis; Methodology; Supervision; Writing – review & editing). **Maaike Waanders‐Oude Elferink** (Formal analysis; Investigation; Writing – original draft). **Ilse van Tilborg** (Investigation; Supervision; Writing – review & editing).

## Data Availability

Data are available from the corresponding author upon request.
